# Bis(2-phenyl­ethyl­ammonium) tetra­chloridocobaltate(II)

**DOI:** 10.1107/S1600536811011603

**Published:** 2011-04-07

**Authors:** In-Hwan Oh, Dahye Kim, Young-Duk Huh, Younbong Park, J. M. Sungil Park, Seong-Hun Park

**Affiliations:** a Neutron Science Division, Korea Atomic Energy Research Institute, Daejeon, 305-353, Republic of Korea; b Department of Chemistry, Dankook University, Gyeonggi-Do, 448-701, Republic of Korea; c Department of Chemistry, Chungnam National University, Daejeon, 305-764, Republic of Korea; d Department of Chemistry, Faculty of Liberal Art & Teacher Education, University of Seoul, Seoul, 130-743, Republic of Korea

## Abstract

Crystals of the title compound, (C_6_H_5_CH_2_CH_2_NH_3_)_2_[CoCl_4_], were grown by the solvent-evaporation method. This inorganic–organic hybrid compound exhibits a layered structure in which isolated CoCl_4_ inorganic layers alternate with bilayers of phenylethylammonium cations. Although the inorganic anion is zero-dimensional, the layered structure is stabilized *via* N—H⋯Cl hydrogen bonds. The CoCl_4_ tetra­hedra connect to the cations through N—H⋯Cl hydrogen bonds, building a two-dimensional network extending parallel to (010).

## Related literature

For inorganic–organic hybrids containing tetra­hedral anions, see: Abdi *et al.* (2005 ▶); Huh *et al.* (2006 ▶); Zouari & Ben Salah, (2004 ▶). For low-dimensional magnetism in inorganic–organic perovskite systems, see: de Jongh (1986 ▶); Park & Lee (2005 ▶, 2006 ▶); Depmeier (2009 ▶); Mitzi (1999 ▶). For classification of hydrogen bonds depending on bond lengths, see: Steiner (1998 ▶, 2002 ▶).
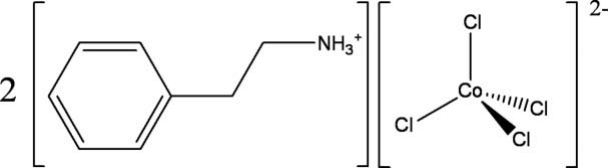


## Experimental

### 

#### Crystal data

(C_8_H_12_N)_2_[CoCl_4_]
*M* *_r_* = 445.10
Monoclinic, 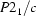

*a* = 7.4623 (13) Å
*b* = 24.664 (3) Å
*c* = 11.1997 (16) Å
β = 91.769 (13)°
*V* = 2060.3 (5) Å^3^
*Z* = 4
Mo *K*α radiation
μ = 1.35 mm^−1^
*T* = 296 K
0.50 × 0.40 × 0.35 mm

#### Data collection

Bruker P4 diffractometer
Absorption correction: multi-scan (*SADABS*; Sheldrick, 1996 ▶) *T*_min_ = 0.237, *T*_max_ = 0.265
4692 measured reflections
3595 independent reflections
1566 reflections with *I* > 2σ(*I*)
*R*_int_ = 0.041
3 standard reflections every 97 reflections  intensity decay: none

#### Refinement

*R*[*F* ^2^ > 2σ(*F* ^2^)] = 0.055
*wR*(*F* ^2^) = 0.161
*S* = 1.03
3595 reflections
211 parameters
2 restraints
H-atom parameters constrained
Δρ_max_ = 0.43 e Å^−3^
Δρ_min_ = −0.30 e Å^−3^

### 

Data collection: *XSCANS* (Bruker, 1996 ▶); cell refinement: *XSCANS*; data reduction: *XSCANS*; program(s) used to solve structure: *SHELXS97* (Sheldrick, 2008 ▶); program(s) used to refine structure: *SHELXL97* (Sheldrick, 2008 ▶); molecular graphics: *PLATON* (Spek, 2009 ▶) and *DIAMOND* (Brandenburg, 1999 ▶); software used to prepare material for publication: *SHELXL97*.

## Supplementary Material

Crystal structure: contains datablocks global, I. DOI: 10.1107/S1600536811011603/si2347sup1.cif

Structure factors: contains datablocks I. DOI: 10.1107/S1600536811011603/si2347Isup2.hkl

Additional supplementary materials:  crystallographic information; 3D view; checkCIF report

## Figures and Tables

**Table 1:** Selected bond lengths (Å)

Co1—Cl4	2.229 (2)
Co1—Cl2	2.251 (2)
Co1—Cl1	2.272 (2)
Co1—Cl3	2.276 (2)

**Table 2:** Hydrogen-bond geometry (Å, °)

*D*—H⋯*A*	*D*—H	H⋯*A*	*D*⋯*A*	*D*—H⋯*A*
N2—H2*C*⋯Cl1^i^	0.89	2.62	3.445 (6)	156
N2—H2*A*⋯Cl4^ii^	0.89	2.51	3.321 (6)	152
N1—H1*C*⋯Cl3^iii^	0.89	2.42	3.291 (8)	167
N1—H1*B*⋯Cl1	0.89	2.55	3.382 (7)	156

Symmetry codes: (i) 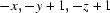; (ii) 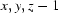; (iii) 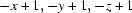.

**Fig. 1.**
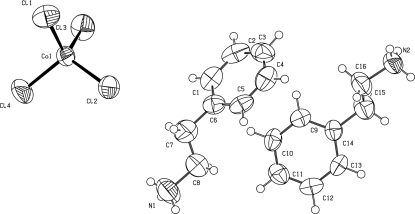
Molecular structure of (C6H5CH2CH2NH3)2CoCl4, showing the atomic labeling and 50% probability displacement elllisoids for non-H atoms.

**Fig. 2.**
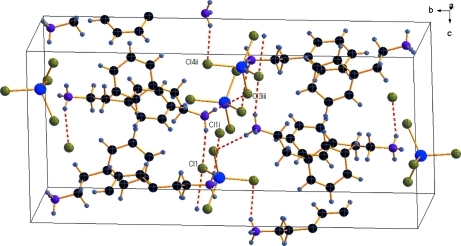
Crystal structure of (C6H5CH2CH2NH3)2CoCl4 viewed along the a axis, showing the N—H···Cl hydrogen bonds as dashed lines.

## supplementary crystallographic information

### Comment

The title compound, (C_6_H_5_CH_2_CH_2_NH_3_)_2_CoCl_4_, belongs to the layered inorganic-organic hybrid systems of general formula A_2_M*X*_4_ (where A = organic cation, *M* = divalent metal, *X* = halides). These systems are of special interest because of typical low-dimensional magnetic systems (de Jongh, 1986; Mitzi, 1999). To investigate the role of interlayer spacing on the magnetic properties, a variety of hybrid systems using long-chain alkylamine have been developed. However, their crystallographic studies are limited because their insolubility make it difficult to obtain a good single-crystal. As a part of our research interest in the low-dimensional magnetism (Park & Lee 2005, 2006), we synthesized a series of the layered inorganic-organic perovskite materials using phenethylamine and present the crystal structure of (C_6_H_5_CH_2_CH_2_NH_3_)_2_CoCl_4_. Among the phenethylammonium-based compounds, several examples with tetrahedral anions are known to literature, for example, (C_6_H_5_C_2_H_4_NH_3_)_2_ZnBr_4_ (Huh *et al.*, 2006), (C_6_H_5_(CH_2_)_2_NH_3_)_2_Cd_0.75_Hg_0.25_Br_4_ (Zouari & Ben Salah, 2004), (C_8_H_12_N)TlBr_4_ (Abdi *et al.*, 2005). Except for (C_8_H_12_N)TlBr_4_, in which the heavy atom has trivalent, the other bivalent compounds have tetrahedral MBr_4_ anions with non-magnetic ions in common. The present paper is the first report of the tetrahedral MCl_4_ with magnetic ion using phenethylamine.

Fig. 1 shows the molecular structure of (C_6_H_5_CH_2_CH_2_NH_3_)_2_CoCl_4_. The asymmetric unit of the title compound consists of two phenethylammonium cations and one isolated CoCl_4_ anion; the latter is arranged as an distorted tetrahedron, whose bond lengths ranging from 2.229 (2) to 2.276 (2) Å (Table 1). Interestingly, the crystal structure exhibits a layered inorganic-organic structure although the dimension of inorganic backbone is 0-dimensional or isolated CoCl_4_ tetrahedra, as shown in Fig. 2. The CoCl_4_ tetrahedral groups are isolated and are connected to the organic cations by N—H···Cl hydrogen bonds *via* the NH_3_-groups. Tab. 2 and Fig. 2 display also the N—H···Cl hydrogen bonds of (C_6_H_5_CH_2_CH_2_NH_3_)_2_CoCl_4_. Between the CoCl_4_ layers, —CNH_3_^+^ ions are located in the space between CoCl_4_ tetrahedra, which is formed by Cl atoms. N—H···Cl hydrogen bonds connect the two groups. The CoCl_4_ tetrahedra connect the C_6_H_5_CH_2_CH_2_NH_3_^+^ ions through hydrogen bonds to build a two-dimensional hydrogen-bonded NH_3_—CoCl_4_ network. Due to the hydrogen bonds, the Co—Cl bond lengths increase, resulting in slightly deformed CoCl_4_ tetrahedra. The obtained bond lengths suggest that the strength of the N—H···Cl hydrogen bonds in the structure can be classified as weak (Steiner, 1998; Steiner, 2002).

### Experimental

CoCl_2_^.^6H_2_O (99%, Aldrich), phenethylamine (C_6_H_5_CH_2_CH_2_NH_2_, 99.5%, Aldrich), HCl (37 wt % in water, Aldrich), and methanol (anhydrous, 99.8%, Aldrich) are used as received. For the preparation of single-crystal (C_6_H_5_CH_2_CH_2_NH_3_)_2_CoCl_4_, 10 ml of a 0.25*M* CoCl_2_^.^6H_2_O methanol solution were mixed with 10 ml of a 0.5*M* phenethylamine methanol solution. 1 mL of an HCl solution was added to the mixed solution. Blue crystals of (C_6_H_5_CH_2_CH_2_NH_3_)_2_CoCl_4_ were obtained after 7 days at room temperature. Elemental analysis of C, H, and N was carried out by CHNS analysis (CE Instrument EA 1112 series). The expected formula of C_16_H_24_N_2_Cl_4_Co was confirmed. The relative weights calculated for C_16_H_24_N_2_Cl_4_Co were: C, 43.17%, H, 5.43%, N, 6.29%; found: C, 43.14%, H, 5.44%, N, 6.23%.

### Refinement

H atoms bonded to C were positioned geometrically and refined based on a riding model (C—H = 0.95Å in aromatic ring and 0.99 Å for CH_2_) with *U*_iso_(H) = 1.2 of their parent atoms. H atoms at N atoms were located in a difference map and refined with distance constrained of N—H = 0.89 Å, and with *U*_iso_(H) = 1.2*U*_eq_(N). C7—C8 and C15—C16 bond lengths were refined with restrained distances 1.545 (2) Å.

### Crystal data

**Table:**

(C_8_H_12_N)_2_[CoCl_4_]	*F*(000) = 916
*M**_r_* = 445.10	*D*_x_ = 1.435 Mg m^−^^3^
Monoclinic, *P*2_1_/*c*	Mo *K*α radiation, λ = 0.71073 Å
Hall symbol: -P 2ybc	Cell parameters from 38 reflections
*a* = 7.4623 (13) Å	θ = 3.3–12.3°
*b* = 24.664 (3) Å	µ = 1.35 mm^−^^1^
*c* = 11.1997 (16) Å	*T* = 296 K
β = 91.769 (13)°	Rectangle, blue
*V* = 2060.3 (5) Å^3^	0.5 × 0.4 × 0.35 mm
*Z* = 4	

### Data collection

**Table:**

Bruker P4 diffractometer	1566 reflections with *I* > 2σ(*I*)
Radiation source: fine-focus sealed tube	*R*_int_ = 0.041
graphite	θ_max_ = 25.0°, θ_min_ = 2.0°
2θ/ω scans	*h* = −1→8
Absorption correction: multi-scan (*SADABS*; Sheldrick, 1996)	*k* = −1→29
*T*_min_ = 0.237, *T*_max_ = 0.265	*l* = −13→13
4692 measured reflections	3 standard reflections every 97 reflections
3595 independent reflections	intensity decay: none

### Refinement

**Table:**

Refinement on *F*^2^	Secondary atom site location: difference Fourier map
Least-squares matrix: full	Hydrogen site location: inferred from neighbouring sites
*R*[*F*^2^ > 2σ(*F*^2^)] = 0.055	H-atom parameters constrained
*wR*(*F*^2^) = 0.161	*w* = 1/[σ^2^(*F*_o_^2^) + (0.0502*P*)^2^] where *P* = (*F*_o_^2^ + 2*F*_c_^2^)/3
*S* = 1.03	(Δ/σ)_max_ = 0.001
3595 reflections	Δρ_max_ = 0.43 e Å^−^^3^
211 parameters	Δρ_min_ = −0.30 e Å^−^^3^
2 restraints	Extinction correction: *SHELXL*, Fc^*^=kFc[1+0.001xFc^2^λ^3^/sin(2θ)]^-1/4^
Primary atom site location: structure-invariant direct methods	Extinction coefficient: 0.0018 (7)

### Fractional atomic coordinates and isotropic or equivalent isotropic displacement parameters (Å^2^)

**Table:**

	*x*	*y*	*z*	*U*_iso_*/*U*_eq_	
Co1	0.25951 (13)	0.52176 (4)	0.77334 (8)	0.0581 (3)	
Cl1	0.0134 (3)	0.55281 (9)	0.67021 (18)	0.0911 (7)	
Cl2	0.2717 (3)	0.55493 (8)	0.96063 (16)	0.0901 (7)	
Cl3	0.5051 (3)	0.54580 (9)	0.67008 (18)	0.0895 (7)	
Cl4	0.2382 (4)	0.43175 (8)	0.78339 (19)	0.1218 (11)	
C1	0.3714 (10)	0.6982 (4)	0.1447 (7)	0.075 (2)	
H1	0.3974	0.6694	0.0946	0.090*	
C2	0.4215 (10)	0.7492 (4)	0.1131 (6)	0.081 (3)	
H31	0.4849	0.7546	0.0439	0.097*	
C3	0.3783 (11)	0.7927 (3)	0.1835 (8)	0.083 (2)	
H3	0.4077	0.8278	0.1608	0.099*	
C4	0.2910 (10)	0.7836 (4)	0.2879 (7)	0.081 (2)	
H4	0.2642	0.8125	0.3376	0.097*	
C5	0.2433 (9)	0.7316 (4)	0.3188 (6)	0.068 (2)	
H5	0.1823	0.7258	0.3888	0.082*	
C6	0.2847 (10)	0.6882 (3)	0.2473 (6)	0.066 (2)	
C7	0.2286 (12)	0.6304 (4)	0.2746 (7)	0.099 (3)	
H7A	0.0987	0.6285	0.2720	0.118*	
H7B	0.2717	0.6067	0.2126	0.118*	
C8	0.2921 (13)	0.6110 (3)	0.3860 (7)	0.106 (3)	
H8A	0.2501	0.6346	0.4484	0.127*	
H8B	0.4221	0.6121	0.3886	0.127*	
C9	0.2308 (9)	0.2503 (3)	0.1306 (5)	0.0560 (17)	
H9	0.2728	0.2687	0.1983	0.067*	
C10	0.2513 (9)	0.1950 (3)	0.1235 (6)	0.0627 (19)	
H10	0.3066	0.1762	0.1866	0.075*	
C11	0.1908 (10)	0.1675 (3)	0.0243 (7)	0.069 (2)	
H11	0.2044	0.1301	0.0198	0.083*	
C12	0.1107 (10)	0.1951 (3)	−0.0676 (6)	0.067 (2)	
H12	0.0702	0.1763	−0.1353	0.080*	
C13	0.0887 (9)	0.2501 (3)	−0.0622 (5)	0.0608 (18)	
H13	0.0334	0.2684	−0.1260	0.073*	
C14	0.1485 (9)	0.2788 (3)	0.0380 (6)	0.0562 (17)	
C15	0.1166 (11)	0.3392 (3)	0.0449 (7)	0.086 (2)	
H15A	0.0752	0.3519	−0.0331	0.103*	
H15B	0.0215	0.3458	0.1002	0.103*	
C16	0.2724 (11)	0.3707 (3)	0.0826 (7)	0.085 (2)	
H16A	0.3704	0.3625	0.0307	0.102*	
H16B	0.3088	0.3602	0.1632	0.102*	
N1	0.2316 (11)	0.5540 (3)	0.4099 (6)	0.115 (3)	
H1A	0.1329	0.5469	0.3661	0.172*	
H1B	0.2083	0.5505	0.4870	0.172*	
H1C	0.3177	0.5309	0.3909	0.172*	
N2	0.2383 (8)	0.4300 (2)	0.0799 (5)	0.0758 (18)	
H2A	0.2107	0.4403	0.0053	0.114*	
H2B	0.3362	0.4475	0.1059	0.114*	
H2C	0.1476	0.4378	0.1267	0.114*	

### Atomic displacement parameters (Å^2^)

**Table:**

	*U*^11^	*U*^22^	*U*^33^	*U*^12^	*U*^13^	*U*^23^
Co1	0.0607 (6)	0.0511 (5)	0.0628 (6)	0.0000 (5)	0.0052 (4)	−0.0041 (5)
Cl1	0.0673 (13)	0.1124 (17)	0.0931 (14)	0.0206 (13)	−0.0035 (11)	0.0131 (12)
Cl2	0.135 (2)	0.0655 (12)	0.0702 (12)	0.0041 (13)	0.0055 (13)	−0.0163 (10)
Cl3	0.0707 (13)	0.1058 (16)	0.0931 (14)	−0.0135 (13)	0.0222 (11)	−0.0084 (12)
Cl4	0.225 (3)	0.0497 (11)	0.0914 (15)	−0.0165 (16)	0.0130 (18)	−0.0131 (10)
C1	0.060 (5)	0.098 (6)	0.066 (5)	0.007 (5)	0.001 (4)	−0.013 (4)
C2	0.048 (5)	0.140 (8)	0.055 (4)	0.009 (6)	0.004 (4)	0.020 (5)
C3	0.068 (6)	0.082 (6)	0.097 (6)	−0.007 (5)	−0.017 (5)	0.030 (5)
C4	0.068 (6)	0.099 (7)	0.076 (5)	0.006 (5)	−0.005 (4)	−0.017 (5)
C5	0.059 (5)	0.104 (6)	0.043 (4)	0.009 (5)	0.007 (3)	0.006 (4)
C6	0.057 (5)	0.077 (5)	0.065 (5)	0.006 (4)	0.000 (4)	0.009 (4)
C7	0.101 (7)	0.099 (7)	0.095 (6)	−0.012 (6)	−0.019 (6)	0.013 (5)
C8	0.130 (9)	0.090 (7)	0.097 (6)	−0.034 (6)	−0.008 (6)	0.013 (5)
C9	0.056 (4)	0.063 (4)	0.049 (4)	−0.012 (4)	0.003 (3)	0.000 (3)
C10	0.062 (5)	0.060 (5)	0.065 (4)	−0.007 (4)	−0.007 (4)	0.019 (4)
C11	0.071 (5)	0.052 (4)	0.085 (5)	−0.007 (4)	0.002 (4)	−0.002 (4)
C12	0.066 (5)	0.083 (6)	0.050 (4)	−0.009 (4)	0.001 (4)	−0.016 (4)
C13	0.061 (5)	0.072 (5)	0.049 (4)	0.000 (4)	−0.009 (3)	0.014 (4)
C14	0.053 (4)	0.051 (4)	0.064 (4)	0.000 (4)	0.001 (4)	0.008 (3)
C15	0.077 (6)	0.066 (5)	0.115 (6)	−0.001 (5)	−0.010 (5)	0.007 (5)
C16	0.089 (6)	0.054 (5)	0.112 (6)	0.003 (5)	−0.011 (5)	−0.003 (4)
N1	0.177 (8)	0.066 (4)	0.103 (5)	−0.025 (5)	0.033 (5)	−0.003 (4)
N2	0.099 (5)	0.050 (4)	0.079 (4)	0.002 (3)	0.005 (4)	0.003 (3)

### Geometric parameters (Å, °)

**Table:**

Co1—Cl4	2.229 (2)	C15—C16	1.451 (9)
Co1—Cl2	2.251 (2)	C16—N2	1.485 (8)
Co1—Cl1	2.272 (2)	C1—H1	0.931
Co1—Cl3	2.276 (2)	C2—H31	0.931
C1—C6	1.358 (9)	C3—H3	0.931
C1—C2	1.363 (10)	C4—H4	0.929
C2—C3	1.376 (10)	C5—H5	0.929
C3—C4	1.374 (10)	C7—H7A	0.970
C4—C5	1.377 (10)	C7—H7B	0.970
C5—C6	1.379 (10)	C8—H8A	0.968
C6—C7	1.519 (10)	C8—H8B	0.969
C7—C8	1.405 (9)	C9—H9	0.930
C8—N1	1.502 (9)	C10—H10	0.930
C9—C10	1.376 (8)	C11—H11	0.929
C9—C14	1.380 (8)	C12—H12	0.929
C10—C11	1.367 (9)	C13—H13	0.930
C11—C12	1.358 (9)	C15—H15A	0.970
C12—C13	1.368 (9)	C15—H15B	0.970
C13—C14	1.389 (8)	C16—H16A	0.970
C14—C15	1.510 (9)	C16—H16B	0.970
			
Cl4—Co1—Cl2	108.41 (8)	C16—C15—C14	114.6 (6)
Cl4—Co1—Cl1	107.67 (11)	C15—C16—N2	112.8 (7)
Cl2—Co1—Cl1	111.10 (9)	H5—C5—C4	119.6
Cl4—Co1—Cl3	110.19 (10)	H2A—N2—H2C	109.5
Cl2—Co1—Cl3	111.65 (9)	H2C—N2—H2B	109.4
Cl1—Co1—Cl3	107.75 (9)	H2A—N2—H2B	109.4
C6—C1—C2	122.0 (7)	C16—N2—H2A	109.4
C1—C2—C3	120.0 (7)	C16—N2—H2A	109.4
C4—C3—C2	119.1 (8)	C16—N2—H2B	109.5
C3—C4—C5	119.9 (8)	H1A—N1—C8	109.3
C4—C5—C6	120.9 (7)	H1B—N1—C8	109.4
C1—C6—C5	118.1 (7)	H1C—N1—C8	109.4
C1—C6—C7	118.9 (7)	H1A—N1—H1B	109.5
C5—C6—C7	123.0 (7)	H1B—N1—H1C	109.5
C8—C7—C6	114.3 (7)	H1A—N1—H1C	109.6
C7—C8—N1	112.5 (7)	C3—C4—H4	119.9
C10—C9—C14	120.6 (6)	C2—C3—H3	120.5
C11—C10—C9	120.3 (6)	C2—C1—H1	119.0
C12—C11—C10	119.6 (7)	H1—C1—C6	118.8
C11—C12—C13	121.0 (6)	C6—C7—H7B	108.5
C12—C13—C14	120.3 (6)	C6—C7—H7A	108.4
C9—C14—C13	118.2 (6)	C7—C8—H8B	108.9
C9—C14—C15	122.0 (6)	C7—C8—H8A	109.0
C13—C14—C15	119.7 (6)		
			
C6—C1—C2—C3	−2.5 (12)	C14—C9—C10—C11	0.2 (11)
C1—C2—C3—C4	2.6 (12)	C9—C10—C11—C12	0.2 (11)
C2—C3—C4—C5	−2.0 (11)	C10—C11—C12—C13	−0.4 (11)
C3—C4—C5—C6	1.1 (11)	C11—C12—C13—C14	0.1 (11)
C2—C1—C6—C5	1.7 (11)	C10—C9—C14—C13	−0.5 (10)
C2—C1—C6—C7	178.3 (7)	C10—C9—C14—C15	177.3 (6)
C4—C5—C6—C1	−0.9 (11)	C12—C13—C14—C9	0.4 (10)
C4—C5—C6—C7	−177.4 (7)	C12—C13—C14—C15	−177.5 (7)
C1—C6—C7—C8	125.0 (9)	C9—C14—C15—C16	49.4 (10)
C5—C6—C7—C8	−58.6 (11)	C13—C14—C15—C16	−132.8 (8)
C6—C7—C8—N1	179.3 (7)	C14—C15—C16—N2	176.0 (6)

### Hydrogen-bond geometry (Å, °)

**Table:**

*D*—H···*A*	*D*—H	H···*A*	*D*···*A*	*D*—H···*A*
N2—H2C···Cl1^i^	0.89	2.62	3.445 (6)	156.
N2—H2A···Cl4^ii^	0.89	2.51	3.321 (6)	152.
N1—H1C···Cl3^iii^	0.89	2.42	3.291 (8)	167.
N1—H1B···Cl1	0.89	2.55	3.382 (7)	156.

Symmetry codes: (i) −*x*, −*y*+1, −*z*+1; (ii) *x*, *y*, *z*−1; (iii) −*x*+1, −*y*+1, −*z*+1.
